#  Modeling Myocardial Infarction in Mice: Methodology, Monitoring, Pathomorphology 

**Published:** 2011

**Authors:** A.A. Ovsepyan, D.N. Panchenkov, E.B. Prokhortchouk, G.B. Telegin, N.A. Zhigalova, E.P. Golubev, T.E. Sviridova, S.T. Matskeplishvili, K.G. Skryabin, U.I. Buziashvili

**Affiliations:** Center “Bioengineering”, Russian Academy of Sciences; Bakoulev Center for Cardiovascular Surgery, Russian Academy of Medical Sciences; Moscow State University of Medicine and Dentistry; The Branch of the Shemyakin and Ovchinnikov Institute of Bioorganic Chemistry, Pushchino, Russian Academy of Sciences; Semashko Railway Hospital

**Keywords:** myocardial infarction, coronary artery, ligation, controlled electrocoagulation, ECG (electrocardiogram)

## Abstract

Myocardial infarction is one of the most serious and widespread diseases in the world. In this work, a minimally invasive method for simulating myocardial infarction in mice is described in the Russian Federation for the very first time; the procedure is carried out by ligation of the coronary heart artery or by controlled electrocoagulation. As a part of the methodology, a series of anesthetic, microsurgical and revival protocols are designed, owing to which a decrease in the postoperational mortality from the initial 94.6 to 13.6% is achieved. ECG confirms the development of large-focal or surface myocardial infarction. Postmortal histological examination confirms the presence of necrosis foci in the heart muscles of 87.5% of animals. Altogether, the medical data allow us to conclude that an adequate mouse model for myocardial infarction was generated. A further study is focused on the standardization of the experimental procedure and the use of genetically modified mouse strains, with the purpose of finding the most efficient therapeutic approaches for this disease.

##  INTRODUCTION 

The simulation of myocardial infarction in animals is of great importance. First of all, there is a need to search for and develop optimal regimens for treating this disease using new approaches, including pharmacological and cell therapies. As a result of the heterogeneity of the concept ‘myocardial infarction’ in humans and its various clinical manifestations (which may occur with another, already present pathology, i.e. diabetes mellitus or an increased concentration of cholesterol in the blood-stream), the question arises as to how to create an animal model which adequately reflects the complex etiology of this disease. The general approach underlying the selection of objects for biomodeling should satisfy the following criteria: 1) convenience in performing open-heart surgeries on animals; 2) the possibility of using genetically modified or selectively bred animal strains with particular features of myocardial infarction simulated; and 3) the possibility of further standardization and certification in compliance with international standards, for both the technology and the laboratory in which the bio-model was obtained. It is reasonable to note that the first two conditions are in conflict with one another since the suitability of organisms for genetic studies is defined by their large population and small size ( *Drosophila melanogaster * and *Danio rerio* are the classical genetic objects). This fact imposes an obvious restriction on carrying out surgical intervention. For to the third condition, it is necessary to obtain a quantity of model animals that will be sufficient in terms of statistical reliability to successfully carry out the first stage of the preclinical testing of new pharmaceutical preparations, or for the performance of cell therapy. From one perspective, the survival rate of animals is dependent upon the reliability of surgery and post-operation recovery protocols. In turn, this makes the process of bio-model development a larger scale one. On the other hand, the process depends on the availability of the certified facilities and hardware required for these procedures in the Russian Federation. Thus, a researcher has to compromise between the size of the animals and availability of their genetic strains, something that could affect the quality of preclinical testing.

In this work, laboratory mice were chosen for the simulation of myocardial infarction. Working with these animals is a well-established procedure at the internationally accredited Research and Production Division Nursery of Laboratory Animals branch of the Shemyakin and Ovchinnikov Institute of Bioorganic Chemistry. Thus, in the case of developing and certifying the technology in accordance with the requirements of the International Organization for Standardization (ISO), there appears to be a possibility of not only using these bio-models in fundamental research, but also supplying Russian and foreign pharmaceutical companies with them. The latter is particularly important, since preclinical testing should be carried out only on standard-model animals with a special health status (specific pathogen-free animals), which is maintained in the nursery at the branch of the Shemyakin and Ovchinnikov Institute of Bioorganic Chemistry. The main advantage provided by mice is that there is a developed network of their genetic resources, something that is in acute shortage in their relatives, rats. The genetic diversity of inbred mouse strains allows us to select animals that are suitable for the study of cardiovascular diseases. In particular, the A/J and C3H/HeJ strains are resistant to atherosclerotic lesions of the aorta occurring when the animals are kept on an atherogenic diet (1.25% cholesterol, 0.5% cholic acid, and 15% fat), while the C57BL/6J mouse strains are extremely sensitive to the atherogenic diet, and the CBA/J strains are partially resistant [1–3]. There are also inbred rat strains which are potentially suitable for infarction simulation (in this context, spontaneously hypertensive rats (SHR) with an increased blood pressure should be mentioned as a popular animal model) [4]. The main advantage of mouse models is the possibility of genetic manipulation of mouse embryonic stem cells, while this type of technology is still poorly developed for rat cells. Transgenesis, knock-out, and knock-in technologies, which are both tissue-specific and inducible, make it possible to remove and insert genes and sometimes to introduce point mutations into the mouse’s genome.

A researcher can always select the desired genes, their diversity enabling the creation of any genetic anomalies. These anomalies can act as a background for the infarction model. Thus, the microsurgical simulation of infarction can be applied to any available genetic model. For instance, if the infarction has to be simulated against a background of an increased cholesterol level, mice carrying an apolipoprotein E4 gene ( *apoE4* ) should be chosen. Meanwhile, if the infarction has to be obtained against a background of cardiomyopathy, BALB/c or CD-1 mice should be used. The catalog of the Jackson Laboratory (USA) offers 373 strains for cardiovascular disease research; twenty of them are selectively bred inbred strains, while the rest of the strains were obtained by genetic manipulations involving more than 50 genes. Among genetic models of diseases to which the microsurgical simulation of infarction can be applied, the following models can be mentioned: hypo- and hypertension, atherosclerosis, cardiomyopathy, lesion of coronary vessels, various metabolic syndromes, ischemia, hypo- and hypercholesterolemia, and hypo- and hypertriglyceridemia. The combination of infarction simulated by means of microsurgical techniques with genetic models creates the prerequisites for reproducing various human heart diseases in mice and for further use of these animals in the clinical testing of drugs and in cell therapy. In the latter case, the observation of fluorescent-labeled (e.g., green fluorescent protein, GFP) or radioactively labeled (labeled with isotopes) cells injected into the heart (or vessels) is possible. The injected cell material is not rejected by homozygous animals, and the sites of its integration can be easily determined by multi-slice computed tomography, thereby performing the intra-vital monitoring of the efficiency of cell therapy. The obvious shortcomings in the case of mice include the small size of their viscera and the small volume of blood circulation. The use of current methods for developing mouse bio-models leads to a high mortality of laboratory animals [5–9], a phenomena that is due to surgical aggression and loss of blood [10–11]. Therefore, a whole complex of anesthetic, microsurgical, and revival protocols is needed in order to perform a successful surgical intervention. This complex involves tracheal intubation for pulmonary ventilation by means of a medical ventilator, inhalation narcosis, bloodless thoracotomy, ligation of the left-anterior descending coronary artery, procedures for the recovery of animals after surgical modification, and measurement of electrocardiograms (ECG).The methodology and technical equipment in these procedures are considerably different from those used in Russia for surgical operations in rats.

 The purpose of the present work is to develop for the first time in Russia a minimally invasive method for simulating myocardial infarction in laboratory mice. This will be accompanied by a complex of postoperation procedures aimed at achieving successful rehabilitation of the animals operated upon, and monitoring their state during the postoperation period. 

##  EXPERIMENTAL 

**Preoperation Period and Narcotization**

**Fig. 1:**
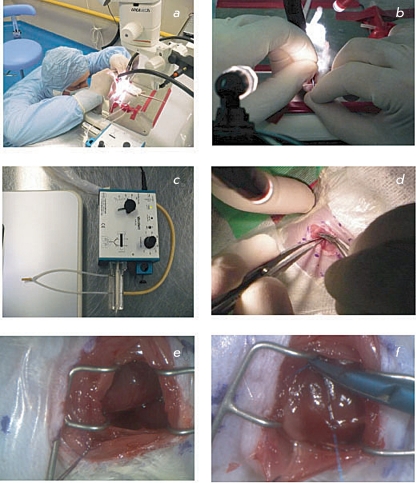
Preoperation and operation procedures during simulation of myocardial infarction in mice: *a – * fixation of a mouse on a heated operation table; *b* – intubation of trachea; *c –* device for artificial pulmonary ventilation “MiniVent Ventilator for Mice”; *d* – incision in the 4-th left intercostal space; *e* – front (lower) surface of the heart can be seen in the incision wound; *f* – ligation of the descending branch of the coronary artery.

**Fig. 2:**
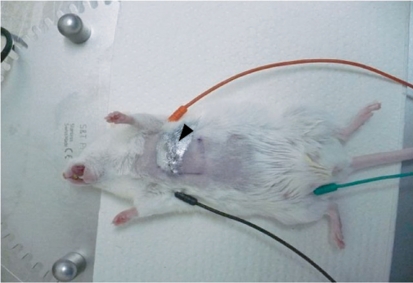
Illustration of postsurgery ECG measurements. A mouse is placed on a heated operation table and connected to the ECG apparatus by three electrodes: one on the left foreleg (red), one on the right foreleg (black), and one on the right hind leg (black). The sutured wound is covered with an AluSpray suspension (shown with an arrow).

In this work, we used 48 CD-1 mice of mixed sex, more than 8 weeks old, and weighing 3,2­36 g. Twenty-four hours prior to the operation, the mice were put into a cage with clean litter and water. Food was completely removed from the feedboxes. Just prior to the operation, the animals were weighed, and the needed volume of anesthetic was calculated. For the anesthesia, the injection mixed zoletil/xylazine narcosis (40–50 mg zoletil (tiletamine + zolazepam) per 1 kg of body weight and 15­–20 mg xylazine per 1 kg of body weight) was used. After surgical anesthesia and prior to tracheal intubation, the official drug, Vetranquil 1% inj. (Ceva Sante Animale, France), containing 1% acepromazine and 0.5% chlorobutanol, was used in a dose of 2–4 mg/kg acepromazine and 1–2 mg/kg chlorobutanol for neuromuscular relaxation when needed. In order to prevent drying of the cornea, a moisture-donating gel, Normlgel – 0.9% (Mölnlycke Health Care), was applied to the eyes of the anesthetized animals prior to the surgery. The preparation of the surgical site on the left side of the chest was performed according to the following procedure: the hair was shaved off, the skin was disinfected with a disinfectant, Decosept (BORER CHEMIE AG, Switzerland), and draped in a sterile transparent adhesive film, Opsite incise drape (Smith & Nephew, England) ( *Fig. 1a* ). Immediately after narcotization of the mice, ECG measurements were performed (the procedure is described below). In order to carry out the tracheal intubation, the mouse was fixed in a supine position on a heated surgical table. The tracheal intubation was carried out using an endotracheal tube with the following dimensions: the outer diameter was 1–1.2 mm, the inner diameter was 0.6–0.8 mm, and the length was 25–30 mm) ( *Fig. 2b* ). The endotracheal tube was connected to a MiniVent Ventilator for Mice (HSE Harvard, Germany) ( *Fig. 1c* ), the inspiratory oxygen concentration was equal to 30% (oxygen was generated using a NewLife oxygen concentrator (AirSep, USA). The calculated values of the volume and the ventilation rate are listed in *Table* .

**Surgical Procedure**

**Table 1:** 

Weight of a mouse, g	Stroke volume, µl	Ventilation rate, strokes/minute
22–27	175	130
28–35	200	120
≥35	225	110

Using a Leica MZ7.5 high-performance stereomicroscope (the magnification range is from 10x to 25x, the working distance is 25–30 cm), an incision in the fourth left intercostal space was made and the muscles of the chest wall (the broadest muscle of the back, the serratus ventralis muscle, and the external abdominal oblique muscle) were prized apart with scissors. Ligatures (surgical silk 3-0) were applied to these groups of muscles, and the muscles were moved in opposite directions. Using an eye dressing, forceps with angled serrated tips (Fine Science Tools, USA) and scissors, the intercostal muscle and parietal pleura were cut. Then, a Mini-Goldstein 3x3 (Fine Science Tools) retractor was placed into the wound. Next, the pericardium was cut by careful blunt dissection, and the lung was moved to the edge of the wound, giving access to the front (lower) surface of the heart ( *Fig. 1e* ). By manipulation with forceps and the pericardium in the incision wound, the heart was brought to a position suitable for finding the left coronary artery. This artery has a vivid red color and pulsates. With the help of an atraumatic needle and a nonabsorbable suture Prolene 6/0 (Ethicon, USA), the coronary artery was ligated in the center of its descending branch ( *Fig. 1f* ). If the ligation is performed correctly, the tachycardia, arrhythmia, and myocardial anemia develop fast in the ligated site. Then, the pericardium and the lung were moved back.

 Since the identification of the coronary artery and its ligation are rather complicated procedures from a technical point of view, we proposed another method for the simulation of myocardial infarction. This method involves producing a necrotic zone by means of an electro-coagulator. In this method for simulating infarction, an active electrode of the electro-coagulator acts directly on the myocardium after the front (lower) surface of the heart is reached. 

After the surgical procedures were performed, the ribs and the pleura were sutured with two or three isolated, interrupted sutures using a VICRYL 5-0 (Ethicon) thread, and the incision wound in the chest wall was closed. Then, the dissected muscles were put together and tightly pressed against each other. The wound was irrigated with a sterile saline solution. After that, an uninterrupted glover’s suture was placed using an atraumatic needle and a nonabsorbable suture VICRYL 4-0 (Ethicon). During this procedure, the chest in the area of the wound was compressed by forceps with the purpose of removing air and creating negative pressure in the thoracic cavity. In order to protect the surface of the surgical wound, the skin was treated with a special suspension of microporous aluminum AluSpray (Vetoquinol, France), which forms a thin layer of aluminum coating on the skin’s surface ( *Fig. 2* ).

**Temperature Conditions**

 In order to prevent the hypothermia that may result as a consequence of narcotization, the animal was placed on the heated surgical table with a heating temperature of 32–34°C. Immediately upon completion of the surgery, the animal was put into a recovery chamber Warm Air System (Vet Tech Solutions, England), supplied with heated (30­–32°C) oxygen-enriched air. 

**Postoperation Period**

 Xylazine in a quantity of 10 mg xylozane per 1 kg of body weight was used for postoperation analgesia in the mice. When an animal emerged, it was put into an individual cage. Moist feed was given to the animal. Normally, there is no need for the removal of absorbable sutures. 

**ECG Measurements**

ECG plays an important role in the diagnostics of myocardial infarction. The changes observed in the ECG allow one to determine the infarction location, its extent and depth; i.e., whether it is large-focal, small-focal or intramural, sometimes the prescription (during the first few weeks), and a number of other features. Therefore, ECG measurements were performed in mice before and after the ligation of the coronary heart artery and coagulation in order to obtain an experimental myocardial infarction. Prior to ECG recording, a mouse was subjected to injection narcosis using a zoletil/xylazine solution (40–50 mg zoletil (tiletamine + zolazepam) per 1 kg of body weight and 15–20 mg xylazine per 1 kg of body weight). Then, the mouse was put in a supine position on a heated surgical table and connected to three electrodes: one on the left foreleg, one on the right foreleg, and one on the right hind leg ( *Fig. 2* ). The ECG was recorded on a PowerLabSupport apparatus (ECG Analysis Module) (ADINSTRUMENTS, Australia). The value of electrocardiographic waves, their duration and intervals were defined in accordance with the parameters proposed in the ECG Analysis software. The excitation and repolarisation width was 100 ms (Pre-P was 10 ms, PR was 50 mc, and RT was 40 ms). The ECG was recorded prior to the surgery, 5–10 min after the surgery, and on the 3rd, 10th, and the 30th days after the surgery.

**Morphological Examination**

 A morphological examination was performed in 32 mouse hearts: in 21 hearts, myocardial infarction was simulated by ligating the left coronary artery; and in 11 hearts, by electrocoagulation of a myocardial site. After their removal, all organs were placed into 10% neutral formalin for 1 day, with the purpose of fixation. According to the macroscopic examination, the hearts had rather similar sizes and weights: the average weight of a mouse heart was 4 g, and the average size was 1.6 × 0.9 × 0.7 cm. In 11 cases, the necrosis was induced by controlled electro-coagulation; a spot trace from the electrode was detected on the epicardium of the left ventricle during macroscopic examination. 

 For histological examination, each heart was cut into two pieces along the interventricular and interatrial septa. These pieces were subsequently held in formalin at room temperature for 24 h with the purpose of further fixation. The pieces were then washed with running water for 1–2 h. The washed pieces dehydrated at room temperature using several portions of solutions with increasing concentrations: once with 70.96 ethanol and twice with 100% ethanol (for 2 h). The pieces were then prepared to be embedded in paraffin in accordance with the following procedure: the pieces were placed into an alcohol/xylene mixture (1 : 1) for 2 h at room temperature and held in a thermostat in a mixture of hot paraffin and xylene (1 : 1) for 2 h at 60°C. The pieces dehydrated and coated with paraffin were placed into metal frames with the dimensions 2 × 2 × 2 cm and embedded with a HISTOMIX paraffin medium (Biovitrum, Russia). After cooling, paraffin blocks containing pieces of the heart in bulk were obtained. Thus, for each heart, two paraffin blocks were obtained, each containing the right or left heart segments, and sections of the interventricular and interatrial septa. 

 The purpose of the histological examination was to detect the focus of myocardial necrosis, which required the performance of a series (layer-by-layer) of transmural histological sections with a thickness of 5 µm each throughout the entire thickness of the cardiac walls from each block. The sections were prepared using an ACCU-CUT SRM 200 rotatory microtome (ACCU-CUT, Japan) and placed on slides. On average, 30 histological sections were prepared from each block. Paraffin was then removed from the sections by holding them in a xylene solution for 15 min and treating with ethanol solutions with decreasing concentrations (twice with 100% ethanol (for 3 min each time), and twice with 96% ethanol). After the removal of paraffin, the sections were washed with distilled water and stained with Mayer’s hematoxylin and eosin. The stained sections were covered with polystyrene and placed under a cover glass. 

##  RESULTS AND DISCUSSION 

**Results of the Surgical Stage**

**Fig. 3:**
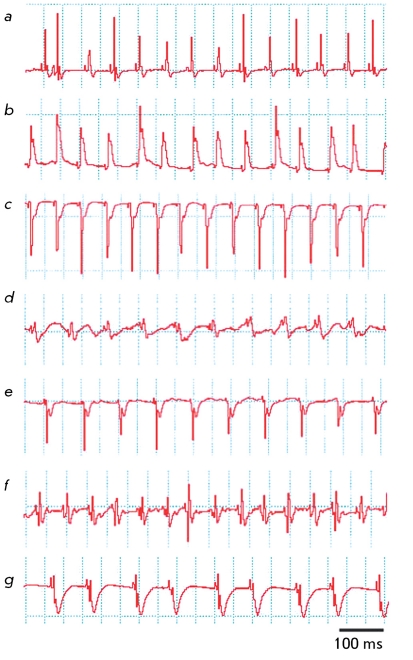
ECG recorded in animals after surgery: *a* – ECG recorded prior to surgery; *b* – impaired heart rhythm in 5–10 minutes after ligation of the coronary artery; *c* – development of a large-focal infarction 10 days after the ligation of the coronary artery; *d* – heart rhythm measured 5 minutes after thermal coagulation of the coronary artery; *e* – development of surface infarction 10 days after thermal coagulation; *f* – tachycardia developed 5–10 minutes after the ligation of the coronary artery; *g* – brachycardia developed on day 3 after the ligation of the coronary artery. The excitation and repolarization width is 100 ms.

 During the first stage of the myocardial infraction simulation, 16 laboratory mice were operated upon. The ligation of the coronary artery was performed in 12 of them, and the controlled electro-coagulation procedure was carried out on four mice. The ECG was recorded on four mice. The mortality rate for this group was 94.5%. Further, the animals from this group were not included into the investigation and considered as a test group. 

 After the procedure was standardized, surgeries were carried out in 32 animals (the ligation of the coronary artery was performed in 21; and coagulation, in 11). Let us note the important details and peculiarities typical to the ligation procedure. At all stages of the surgery described above, it is extremely important to follow the topographic anatomic reference points. Otherwise, uncontrolled bleeding or damage to lung tissue could occur; this is inadmissible, since it leads to the death of laboratory animals. After opening the pericardium, the anterolateral wall of the myocardium with the left coronary artery in it should be identified. Since the coronary artery has a small diameter, its identification is complicated. The left descending branch of the coronary artery is characterized by an opalescent white band following a short oblique direction relative to the main direction of the operation procedure. When the artery was identified, a synthetic suture Prolene 6/0 in an autraumatic needle was brought under it. The artery was ligated, a microsurgical knot was formed, and the suture ends were cut using microsurgical scissors. During the ligation stage, it is extremely important to control the thickness of the tissue taken; if the needle is inserted too deeply, the probability of myocardial perforation is high; which leads to immediate death. The site the ligature is applied to should be determined correctly, since if it is applied not to the descending branch but to the main trunk of the left coronary artery, extensive myocardial infarction incompatible with life occurs. In order to determine which suture material is optimal for this operation, a comparative study of different types of suture material was performed. Synthetic monofilamental nonabsorbable material demonstrating no sawing effect was selected; Prolene 6/0 suture is an example of this kind of material. 

 The average surgery time for this group of animals was 57 min (39–75 min). Post-operation lethality decreased to 13.6% against the 94.6% observed in surgery in mice from the test group. These results are in agreement with the published data and in line with the task set. 

**ECG Data**

In most mice with myocardium infarction simulated by ligating the coronary artery, rhythm disturbance, i.e. atrial fibrillation, was observed after the surgery. On the 10th day after the surgery, ECG typical for large-focal myocardial infarction was obtained; this type of infarction is characterized by the disappearance of the R wave, emergence of a broad and deep QS complex, and lifting of the ST segment above the isoelectric line ( *Figs. 3a–3c* ). The formation of a large-focal myocardial infarction was also observed in mice on the 3rd and 9th day after coagulation. In some cases, coagulation resulted in the formation of the surface myocardial infarction ( *Figs. 3d, 3e* ). In the case when the coronary artery was ligated, the emergence of tachycardia (pathological Q and a decrease in the R voltage) causing bradycardia against a background of myocardial infarction was revealed ( *Figs. 3f, 3g* ).

**Results of Histological Examination**

**Fig. 4:**
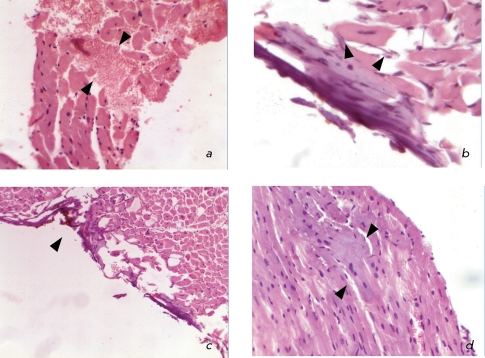
Histopathology of the hearts in the case of infarction simulated in mice: *a* – beginning of coagulational necrosis and cardiomyocytes disorganization, stromal edema, and haemorrhage; hematoxylin and eosin staining; magnification of 20x; *b* – lack of cross striation within the sarcoplasm of cardiomyocytes; apoptotic patterns; hematoxylin and eosin staining; magnification of 60x; *c* – necrosis stripe of the epicardium and surface cardiomyocytes occurring under the impact of the electrode during coagulation; edema of the myocardial interstitium; hematoxylin and eosin staining; magnification of 10x; *d* – fragmentation of muscle fibers as a result of fibrillation; hematoxylin and eosin staining; magnification of 40x.

The histological examination revealed foci of necrosis in 28 mice (87.5%), whereas no necrosis was observed in 4 mice (12.5%). The mice with no necrosis belonged to the group of mice in which myocardial infarction was simulated by ligation of the coronary artery. This fact can in all likelihood be attributed either to the individual characteristics of heart blood supply with a well-developed collateral system or to an error in the method. A great number of types of heart blood supplies are known in humans, since the development of the coronary arteries varies widely. Consequently, the contribution of each to the blood supply of the left and right ventricles and atria may vary for each particular case, as well. In addition to the main left coronary and right coronary arteries, the relative weight of which is 15% (according to Smolyannikov and Naddachina [11]), a number of other forms of blood supply of the heart exist. Therefore, it can be assumed that similar features of blood supply are present in the mouse heart. Thus, the ligation of the coronary artery might have had no effect on the development of myocardial infarction in four cases.

When myocardial infarction was simulated by ligation of the coronary artery, the emergence of small scattered foci of coagulation necrosis in cardiomyocytes and their disorganization and the occurrence of stromal edema and hemorrhage without inflammatory reaction ( *Fig. 4a* ) were observed in the form of necrosis of individual cells. These changes manifested themselves in the disappearance of cross striation within the sarcoplasm of cardiomyocytes and the emergence of apoptosis. The changes were found in different parts of the left ventricle ( *Fig. 4b* ).

In the case of controlled electro-coagulation, a direct coagulation necrosis of the epicardium and a layer of cardiomyocytes were observed in the site to which the electrode was applied ( *Fig. 4c* ). In deeper layers of the myocardium, fragmentation of muscle fibers was observed; a histological sign of the presence of intravital fibrillation of the left ventricle and edema of the myocardial interstitium ( *Figs. 4c, 4d* ). The fibrillation of ventricles is most likely associated with the direct impact of the electrode on the myocardium and with the secondary ischemia induced by the damage to the local coronary blood flow. These sites can undergo necrotic changes with time, so that the necrotic zone can expand.

 It should be taken into account that from the pathomorphological point of view, infarction is a form of necrosis that occurs as a result of the absolute or relative insufficiency of arterial blood circulation in organs that have no access to oxygen. Since in the case of electro-coagulation the necrotic changes emerge mostly due to the direct impact of the electrode, it is unreasonable to consider these changes as an actual myocardial infarction. Myocardial infarction is based on the coagulation (dry) necrosis of cardio-myoctes occurring upon hypoxia and developed tissue acidosis. Coagulation necrosis develops in tissues with a low fluid content, a high concentration of proteins, and a low activity of hydrolytic enzymes. Another form of cell death observed in the investigated material is apoptosis. 

 Apoptosis is genetically programmed cell death that is normally found in various organs and tissues, especially in those characterized by the constant renewal of cells. This process is a result of the activation of particular genes under the action of various stimuli. Apoptosis removes the unneeded cells that complete their cycle during the following processes: embryogenesis, homeostatic regulation of maintenance of the cell population in tissues, immune protection, aging, and removal of cells damaged by various pathologic factors. In particular, hypoxia may cause apoptosis if oxygen deficiency is not critical, whereas apparent oxygen starvation results in cell necrosis. Histologically, the nucleus undergoes the most significant changes during apoptosis. Chromatin condensates into compact aggregates of various shapes and sizes (karyopyknosis), which manifests itself under a light microscope as an irregular shape and hyperchromicity of a nucleus. A similar situation was observed when simulating myocardial infarction by ligating the coronary artery. Further, during apoptosis, the nucleus undergoes fragmentation (karyorrhexis) and cytoplasm disintegrates into several linked apoptotic bodies due to the formation of membrane bridges. Some fragments may contain no nuclear material at all. Finally, apoptotic bodies are phagocytized by surrounding healthy cells or tissue macrophages. Unlike necrosis, apoptotic bodies do not cause any inflammatory response, since the integrity of the cell’s membrane persists until phagocytosis, which prevents the excretion of cell enzymes into surrounding tissues and does not result in chemotaxis for inflammatory cells. 

 Thus, the morphological patterns of changes in mice in which infarction was simulated by ligating the left coronary artery and that in the mice with infarction induced by controlled electro-coagulation are different. In the first group, they were characterized by the presence of small scattered foci of coagulation necrosis and foci of apoptosis in different sites of the left ventricle occurring due to a large area of tissues subjected to hypoxia. The insignificance of necrotic foci in the case when the coronary artery is ligated is most likely due to the well-developed network of blood supply and the absence of atherosclerotic lesions of arteries. In the second group of mice, local myocardial damage emerged mostly due to the direct damage done to the tissue. According to the conventional stages of myocardial infarction used in the morphology of this disease, the histological findings showing the incipient coagulation necrosis of cardio-myocytes, the edema and the hemorrhage without inflammatory reaction correspond to the prescription of a myocardial infarction of about 4–12 h. Irreversible changes in the myocardium at the occurrence of critical ischemia begin even after 0.5 h; however, they can be observed neither macroscopically nor histologically at this stage and can be detected only by electron microscopy. These changes are revealed during histological examination only after 4 h. Twelve hours after the occurrence of the infarction, necrotic changes involving karyorrhexis in the cell intensify and the first leukocytes emerge. After 24 h, an apparent necrosis of cardiomyocytes and interstitial infiltration of neutrophils emerge. On the fourth day, the phagocytosis of dead cardiomyocytes begins, and the granulation tissue appears at the margins of the infarction by the tenth day. Two weeks after the infarction, the infarction zone is completely replaced by granulation tissue and collagen appears. During the next six weeks, cicatrical tissue is formed. The changes observed in the first group of hearts correspond to the early stage of myocardial infarction. 

##  CONCLUSIONS 

 The results obtained in this work allow to conclude that optimal anesthesia for surgery on simulating myocardial infarction is injecting zoletil/xylazine narcosis with muscle relaxants, accompanied by mechanical ventilation. Choosing the proper temperature conditions during pre-, intra- and post-operation periods allow to minimize the death rate of animals associated with nonsurgical causes. Accurate compliance with the specified anatomo-topographic landmarks during the stage of thoracotomy and pericardiotomy allows to minimize the intra-operative hemorrhage and the probability of lung tissue damage. Prolene 6/0 was chosen as an optimal suture material for ligating the descending branch of the left coronary artery. According to ECG data and postmortem histological examination, the model proposed for simulating a myocardial infarction is adequate; however, it requires further investigation for the purpose of standardization, in line with international standards. 
